# Active transportation and bullying in Canadian schoolchildren: a cross-sectional study

**DOI:** 10.1186/s12889-015-1466-2

**Published:** 2015-02-07

**Authors:** Ioana Cozma, Atif Kukaswadia, Ian Janssen, Wendy Craig, William Pickett

**Affiliations:** Department of Public Health Sciences, Queen’s University, 99 University Avenue, Kingston, ON K7L 3N6 Canada; School of Kinesiology and Health Studies, Queen’s University, Kingston, ON Canada; Department of Psychology, Queen’s University, Kingston, ON Canada; Clinical Research Centre, Kingston General Hospital, Kingston, ON Canada

**Keywords:** Active transportation, Adolescent, Bullying, Child, Health behaviours, Health promotion, Physical activity

## Abstract

**Background:**

Bullying is a recognized social problem within child populations. Engagement in childhood bullying often occurs in settings that are away from adult supervision, such as en route to and from school. Bullying episodes may also have a negative impact on school childrens’ decisions to engage in active transportation.

**Methods:**

Using a cross-sectional design, we analyzed reports from the 2009/10 cycle of the Canadian Health Behaviour in School-Aged Children (HBSC) study. Records from this general health survey were obtained for 3,997 urban students in grades 6–10 who lived in close proximity of their school and were hence ineligible for school bussing. Students who indicated walking or bicycling to school were classified as engaged in active transportation. Victims and perpetrators of bullying were defined using standard measures and a frequency cut-off of at least 2–3 times per month. Analyses focused on relations between bullying and active transportation, as well as barriers to active transportation as perceived by young people.

**Results:**

27% of young people indicated being victimized, and 12% indicated that they engaged in bullying. Girls were more likely to be victimized than boys, and younger students were more likely to be victimized than older students. Engagement in active transportation was reported by 63% of respondents, of these, 68% indicated that worrying about bullying on the way to school was an impediment to such transportation methods. Victimization by bullying (adjusted OR = 1.26, 95% CI: 1.00 – 1.59) was reported more frequently by children who used active transportation.

**Conclusions:**

Health promotion efforts to promote engagement in active transportation of students to school have obvious value. The potential for modest increases in exposure to bullying should be considered in the planning of such initiatives.

## Background

Bullying has been recognized as an important problem among child populations internationally [1]. Bullying can be defined in various manners, with a traditional definition being “a deliberate, repeated or long-term exposure to negative acts performed by a person or group of persons regarded as having a higher social status than the victim”, [1] and another “acts of using direct or indirect physical and verbal tactics to distress or control another” [2]. In a recent cross-national study of mainly European and North American countries, reports of recent engagement in bullying behaviours among young people aged 11–15 years ranged from 9% to 13% for victimization, and 8% to 12% for perpetration [3]. Dependent upon the age group under study, up to one-third of youth reported recent victimization due to bullying, with reported proportions highest within the youngest age groups [1]. These may be underestimates: a recent systematic review summarized the prevalence of bullying across 80 studies that reported rates for cyber and traditional perpetration, cyber and traditional victimization, or both among adolescents [4]. Mean prevalence rates were 35% for traditional bullying involvement and 15% for cyber bullying.

Adult supervision is a major protective factor for all forms of child bullying [5]. Compared to same-age peers, adults are more likely to intervene in bullying episodes [6]. Lower levels of adult supervision and inadequate monitoring are therefore related to increased risks for aggressive behaviour among young people. Settings chosen for bullying episodes include locations where children transport themselves to and from school. Approximately 30% of youth report being victimized by bullying in such settings, [7] and efforts to bolster supervision have been made in interventions programs as a means to reduce bullying in school facilities, on school grounds during break periods, on school buses, and on routes where children are transported to and from schools.e.g., [6,8].

Reported engagement by students in active forms of transportation, such as walking and cycling to school, are declining in North America [9,10]. This is a cause for concern as these methods of transportation are forms of physical activity that can contribute to improved health [10]. Boys who engage in active transportation to school have a lower body mass index, are fitter, and engage in more physical activity compared to those who did not [10]. While programs exist to encourage active transportation practices, [11] fears of violence and worries about bullying may reduce participation, because children and their parents are afraid of verbal, physical and relational attacks in unsupervised contexts when they are transporting themselves to school [12,13]. In addition, associations between engagement in active transportation and heightened experiences with bullying, however, have rarely been studied and not been verified in population-based studies.

To address these gaps in the public health literature, we had a unique opportunity to examine bullying as a potential consequence of active transportation in a national sample of Canadian school children. Our hope was that evidence from this study might contribute to health promotion efforts aimed at the optimization of health in populations of young people.

## Methods

### Data source and participants

Analyses were based on the sixth (2009/10) Canadian cycle of the Health Behaviour in School-Aged Children (HBSC) Study [14]. HBSC is a World Health Organization affiliated, cross-national study that involves study of health behaviours, their potential determinants and their consequences among 11–15 year olds. It employs an international mandatory questionnaire with additional modules that are used by sub-samples of participating countries [15]. In the sixth cycle in Canada, in addition to data obtained from individual students, geographic measures were obtained to describe characteristics of school neighborhoods, as well as each student’s travel patterns and distances from home to school.

The Canadian version of the 2009–10 survey was completed by 26,078 students (77% of eligible participants) from 8 provinces and the three northern territories (11 of 13 eligible jurisdictions) [16]. Schools were sampled with replacement; when a school was unable or unwilling to participate a neighbouring school with similar demographic characteristics was selected for study. The current analysis was restricted to urban students (n = 3,997) for which there was clear evidence that they lived proximally (within 1.6 km) to their school as assessed by postal code centroids or commuting times. This inclusion criterion limited the study population to students who were most likely ineligible for transportation in school buses. Consent for participation in the study (active or passive as dictated by local school board customs) was obtained at three levels – school boards, school principals, and parents/guardians. The HBSC study protocol was approved by the General Research Ethics Board of Queen’s University and the Health Canada and Public Health Agency of Canada Research Ethics Board. The current analysis received subsequent approval from the Queen’s University Health Sciences Research Ethics Board.

### Key measures

#### Active transportation (exposure variable)

Participants who indicated “walking” or “bicycling” as their main method of transportation to school were classified as engaged in active transportation. Those reporting that their main method of transportation to school was by “bus”, “train”, “streetcar”, “subway”, “boat/ferry”, “car”, “motorcycle”, “moped”, or “other” forms of transportation were classified as not being engaged. The items used to measure active transportation (type and duration) had been tested previously [17,18]. The first item (identifying the type of transportation used to arrive at school) had a high reported level of agreement between participants’ reports (Cronbach’s alpha ≥0.80) [17]. The second item (identifying the duration of transportation to school) has also been tested psychometrically (percentage agreement range 74%-96%) [18].

#### Bullying (outcome variables)

Students were classified as being perpetrators or victims of bullying, categorized in a dichotomous fashion (yes or no) based upon a threshold frequency of the behaviour at least “2-3 times per month”. This classification was applied to an overall measure of bullying and for each of four specific subtypes (verbal, relational, physical, and cyber). *Verbal bullying* was defined as hurtful teasing delivered verbally. *Relational bullying* has the intended effect of ostracizing an individual from a group, possibly achieved through rumour spreading or social exclusion. *Physical bullying* referred to the use of physical means to assert dominance over an individual, (i.e., hitting, kicking, shoving). Lastly, *cyber bullying* involved Internet use in order to control an individual, (i.e., a composite question that addressed: someone sent mean instant messages, wall postings, emails and text messages, or created a Web site that made fun of me). These bullying items have been tested for face validity and reliability; the questions identifying involvement in most specific types of bullying were developed and evaluated originally by Olweus [1,19]. Questions used to measure the frequency of bullying involvement represented variations of the original questionnaires and were found to produce results that were congruent with the Olweus Bullying Victimization Questionnaire [20].

In addition to the above bullying module, we also used an additional item, collected for descriptive purposes, to determine whether “worrying about being bullied or attacked” was an impediment to active transportation to school” (yes or no). This was one of several questions used in the HBSC that documented facilitators and potential barriers to engagement in active transportation.

#### Covariates

Possible confounders of the relationship between exposure to active transportation and victimization by, and separately, perpetration of, adolescent bullying were documented based upon the extant literature. Variables that had been identified as risk factors for bullying and also met classic statistical criteria for confounding [21] were retained in our statistical models. Confounders available for study therefore included: gender (*male or female*) [2,22] age (*in years*), [2,21] adiposity (*body mass index categorized using age and gender-specific cut-points for normal weight, overweight and obese*), [23,24] engagement in arguments with parents, (5 response options of “strongly agree” through “strongly disagree”) [25] communication with fathers and mothers (*how easy is it for you to talk to the following persons about things that really bother you?;* 5 response options of “very easy” through “don’t have or see this person”), [26] parental trust (*my parents trust me;* 5 response options of “strongly agree” through “strongly disagree”), [27] neighbourhood trust (*you can trust people around here*; 5 response options of “strongly agree” through “strongly disagree”), [28] sense of belonging at school (*I feel I belong at this school;* 5 response options of “strongly agree” through “strongly disagree”), [29] and support from teachers (*I feel a lot of trust in my teachers*; 5 response options of “strongly agree” through “strongly disagree”) [30].

### Statistical analysis

We performed analyses using SPSS version 21.0 (IBM Corp., Armonk, NY). We first characterized the sample demographically. Patterns of bullying and its specific types (victimization then perpetration) were then described by gender and school grade. Tests for statistical significance of observed group differences were performed. Similar analyses were conducted for reports of active transportation, then also potential barriers to active transportation. We then used multiple logistic regression to examine relations between engagement in active transportation and reports of bullying, both as a victim and then as a perpetrator. We examined overall (any bullying) then specific bullying outcomes in these analyses. These analyses were viewed as exploratory. Confounder retention during the modeling process was informed by past literature, backwards elimination (a liberal p-value of 0.15 for inclusion), and a change in odds ratio of 10% or greater between unadjusted and adjusted models.

## Results

Following exclusions, the total sample available for analysis was 3,997 students (Table 1). Approximately equal numbers of boys and girls were studied and the majority of students were in grades 6–8. More students perceived themselves to be affluent than not, and the vast majority of participants indicated that their family owns at least one car.

**Table 1:** **Demographic characteristics of the study population of young urban Canadians living within 1 mile of their school (n = 3,997)**

**Characteristic**	**N**	**%**
**Gender**		
Male	1930	48.3
Female	2067	51.7
**Age**		
≤12	808	20.2
13	927	23.2
14	903	22.6
15	633	15.8
≥16	711	17.9
Missing	15	0.4
**Grade**		
6-8	2759	69.0
9-10	1238	31.0

In total, 27% of the participants reported at least one type of victimization by bullying (Table 2). Boys and girls reported similar prevalence levels. Girls were more likely to be targeted by relational and cyber types of victimization, whereas boys were more likely to be targeted by physical victimization. Reported victimization levels declined with increasing grade level. Younger students were more likely to be victimized verbally, relationally, and physically. Boys were more likely to engage in perpetration overall, and specifically using verbal and physical means. Older students exceeded younger students’ involvement in perpetration overall and within all subcategories with the exception of physical perpetration.

**Table 2:** **Percentages of victimization and perpetration of bullying among young urban Canadians living within 1 mile of their school**

	**Total**		**By Gender**					**By School Grade**				
			**Boys**		**Girls**			**≤ Grade 8**		**≥ Grade 9**		
	**N**	**%**	**N**	**%**	**N**	**%**	**p-value**	**N**	**%**	**N**	**%**	**p-value**
**Victimization**	**3924**	**100.0**	**1890**	**100.0**	**2034**	**100.0**	-	**2704**	**100.0**	**1220**	**100.0**	-
**Any**	**1069**	**27.2**	**491**	**26.0**	**578**	**28.4**	**.087**	**796**	**29.4**	**273**	**22.4**	**< .001**
Verbal	791	20.2	387	20.5	404	19.9	.64	581	21.5	210	17.2	.006
Relational	629	16.0	256	13.5	373	18.3	< .001	484	17.9	145	11.9	< .001
Physical	252	6.4	152	8.0	100	4.9	< .001	208	7.7	44	3.6	< .001
Cyber	193	4.9	70	3.7	123	6.0	.001	131	4.8	62	5.1	.41
**Perpetration**	**3935**	**100.0**	**1897**	**100.0**	**2038**	**100.0**	-	**2714**	**100.0**	**1221**	**100.0**	-
**Any**	**457**	**11.6**	**254**	**13.4**	**203**	**10.0**	**.001**	**285**	**10.5**	**172**	**14.1**	**< .001**
Verbal	362	9.2	215	11.3	147	7.2	< .001	219	8.1	143	11.7	< .001
Relational	192	4.9	98	5.2	94	4.6	.43	123	4.5	69	5.7	.001
Physical	149	3.8	91	4.8	58	2.9	.001	105	3.9	44	3.6	.038
Cyber	88	2.2	49	2.6	39	1.9	.16	53	2.0	35	2.9	.16

Table 3 summarizes the methods of transportation to school reported by the students. Active transportation was reported most commonly (62.6%), followed by motorized/private transportation (24.4%) and public transportation (13.0%). Walking was the preferred form of active transportation. Boys were more likely to engage in active transportation to school than girls.

**Table 3:** **Description of active transportation methods to school among young Canadians living within 1 mile of their school**

	**Total**		**By Gender**					**By School Grade**				
			**Boys**		**Girls**			**≤ Grade 8**		**≥ Grade 9**		
	**N**	**%**	**N**	**%**	**N**	**%**	**p-value**	**N**	**%**	**N**	**%**	**p-value**
**Total Sample**	**3997**	**100.0**	**1930**	**100.0**	**2067**	**100.0**		**2759**	**100.0**	**1238**	**100.0**	
**No Active Transportation**	**1495**	**37.4**	**625**	**32.4**	**870**	**42.1**	**< .001**	**1058**	**38.3**	**437**	**35.3**	**.066**
Public Transportation	520	13.0	222	11.5	298	14.4	.006	368	13.3	152	12.3	.36
Motorized Private Transportation	975	24.4	403	20.9	572	27.7	< .001	690	25.0	285	23.0	.18
**Any Active Transportation**	**2502**	**62.6**	**1305**	**67.6**	**1197**	**57.9**	**< .001**	**1701**	**61.6**	**801**	**64.7**	**.066**
Walking	2421	60.6	1245	64.5	1176	56.9	< .001	1630	59.1	791	63.9	.004
Bicycling	81	2.0	60	3.1	21	1.0	< .001	71	2.6	10	0.8	< .001
**Worrying about being bullied or attacked as an impediment to active transportation**												
Total sample	2691	68.0	1187	62.1	1504	73.5	< .001	2026	74.1	665	54.4	< .001
Those using active transportation	1654	66.7	795	61.5	859	72.3	< .001	1218	72.1	436	55.1	< .001
Those using public transportation	362	71.0	140	64.2	222	76.0	.004	283	78.4	79	53.0	< .001
Those using private transportation	675	69.9	252	63.2	423	74.7	< .001	525	76.8	150	53.4	< .001

When we examined facilitators and barriers to active transportation to school, “worrying about being bullied or attacked on the way to school” was identified as an impediment by 68.0% of the study population, and especially by girls and younger students (73.5% and 74.1%, respectively). Such fears were also reported for those who reporting using other modes of transportation, with 71% who used public transportation reporting such worries, and 69.9% who used other motorized/private modes of transportation.

Table 4 summarizes the potential effects of engagement in active transportation (yes vs. no; considered as the independent variable) on reported victimization by bullying, then perpetration by bullying (considered as the dependent variables). Findings are also presented from logistic regression models describing specific types of bullying behaviours as outcomes. A statistically significant increase in risk was found between reported engagement in active transportation and “any victimization”. This association remained after adjustment for potential confounders. Relations between active transportation and overall perpetration, as well as categories of perpetration (verbal, relational, physical) were consistently positive (unadjusted results). These findings did not remain after adjustment for potential confounders.

**Table 4:** **Results of logistic regression analysis examining relations between engagement in active transportation to school and various types of victimization and perpetration of bullying**

	**Unadjusted**		**Adjusted**	
Victimization*	**Odds Ratio**	**(95% CI)**	**Odds Ratio**	**(95% CI)**
Any				
Other transportation	1.00			
Active transportation	1.25	(1.04 – 1.51)	1.26	(1.00 – 1.59)
Verbal				
Other transportation	1.00			
Active transportation	1.16	(0.94 – 1.42)	1.19	(0.92 – 1.54)
Relational				
Other transportation	1.00			
Active transportation	1.02	(0.81 – 1.28)	0.95	(0.72 – 1.25)
Physical				
Other transportation	1.00			
Active transportation	1.02	(0.72 – 1.44)	0.98	(0.65 – 1.48)
Cyber				
Other transportation	1.00			
Active transportation	1.18	(0.81 – 1.73)	0.92	(0.57 – 1.46)
Perpetration*				
Any				
Other transportation	1.00			
Active transportation	1.24	(0.95 – 1.61)	1.02	(0.76 – 1.38)
Verbal				
Other transportation	1.00			
Active transportation	1.15	(0.86 – 1.54)	0.99	(0.71 – 1.37)
Relational				
Other transportation	1.00			
Active transportation	1.39	(0.90 – 2.13)	1.31	(0.79 – 2.17)
Physical				
Other transportation	1.00			
Active transportation	1.09	(0.70 – 1.69)	0.91	(0.55 – 1.51)
Cyber				
Other transportation	1.00			
Active transportation	0.98	(0.57 – 1.70)	0.72	(0.37 – 1.38)

*Victimization adjusted for the following covariates: (1) gender, (2) grade, (3) fighting with parents, (4) difficulty talking to father, (5) difficulty talking to mother, (6) lack of neighbourhood trust, (7) not belonging at school, (8) parental trust, and (9) body mass index.

*Perpetration adjusted for the following covariates: (1) gender, (2) grade, (3) lack of neighbourhood trust, (4) not belonging at school, (5) parental trust, (6) support from teachers, and (7) body mass index.

## Discussion

This study identified relationships between engagement in active transportation to school and episodes of bullying among young urban Canadians who lived within 1 mile of their school and were not eligible for school bussing. It also documented common and negative perceptions of young people surrounding their risks of being bullied while walking or biking to school. We found that a large percentage of young people (68%; 74% in girls) are worried about being bullied in active transportation environments. If young people or their parents respond to these perceptions by finding other transportation options, such as being driven in a family automobile, this is a key barrier to engagement in active transportation.

There have been considerable recent efforts to increase the engagement of young people in active modes of transportation as a general health promotion strategy [10,11]. Such initiatives have inherent value as one component of a multi-faceted approach to addressing the obesity and physical inactivity challenges that are pervasive in modern society, especially among child populations. Implementation of active transportation strategies need, however, to consider both their positive and negative potential effects. In addition to the positive effects of physical activity, [10,11] past studies have suggested, for example, potential increases in bicycling injury, [17] pedestrian injury, [17] impaired lung function (in adults), [31] as well as episodes of violence [12,13] in association with active transportation. A 2009 systematic review examining environmental correlates of children’s active transportation identified eight studies where parents concerns about child safety (including bullying) influenced decision-making around active transportation; statistically significant declines in engagement in active transportation were identified in 3/8 studies, with the remainder showing no effect [32].

However, the literature suggests that most reported negative effects have been modest in strength, and are counterbalanced by the many positive influences of active transportation on physical health.

Strengths of this analysis include the contemporary importance of the study questions to the physical activity and also general public health literatures. The size and national scope of our urban sample are also notable. We profiled the nature of bullying potentially associated with active transportation in a comprehensive manner, and we view our findings as novel within the Canadian health promotion literature.

The HBSC study is limited by its cross-sectional nature, which suggests the need for caution in the interpretation of the temporality of observed relationships. In the cases were we observed relations between active transportation and bullying, we cannot be definite as to whether the proposed active transportation exposure preceded the bullying outcome, or whether the exposure was a consequence of the outcome (reverse causality). It is also based upon self-reports that are subject to recall error and social desirability biases, especially for behaviours such as bullying that are measured subjectively. Our study also focused on an urban sub-sample of the full national study population with no access to school bussing, and findings might not be generalizable to other jurisdictions and geographic contexts. Because of the nature of this sample, we also did not apply sampling weights during our analyses nor account for the overall clustered nature of the sampling design via design effects or multi-level methods; confidence intervals may therefore be slightly narrower than their true values.

Evidence provided by this population-based analysis does inform the development and refinement of health promotion programs and associated health policy. Our findings suggest that the majority of Canadian young people in grades 6–10 in urban settings who live in close proximity to their school actually do engage in active transportation to school, most often by walking. This is a positive finding - perhaps even one to celebrate. Yet, it is sobering to observe that the majority of young people also report fears about being bullied or attacked as a potential impediment to active transportation, with such fears being higher in more vulnerable groups, those being girls and younger students. This was accompanied by a statistically significant increase in the relative odds of victimization, indicating that these fears are not unfounded. It is important to note, however, that these effects were modest in size and mainly limited to verbal bullying alone. Still, if youth are taking public transportation and family vehicles as an alternative to active transportation due to fear of bullying, alleviation of such concerns through a reduction of bullying could improve participation in active travel. This is in addition to the need to address bullying as an unacceptable social behaviour among young people in general, regardless of the mode of transportation used to transport them to school.

## Conclusion

Our findings suggest that bullying and fear of bullying are pervasive phenomena in urban Canadian settings. Yet, there is little that is unique about such active transportation environments that should enable such behaviours and associated fears that would not present in other settings or for other modes of transportation, with the exception of private vehicle transport. Increases in relative odds of bullying were modest, and limited to a single type of bullying. We conclude that school-based programs and policies should continue to promote active methods of transportation, with guarded consideration and prevention of the potential negative effects of such programs in terms of associated bullying behaviours.

## Abbreviations

CI: Confidence interval
GIS: Geographic information systems
HBSC: Health behaviour in school-aged children survey
OR: Odds ratio
